# Design and protocol of the multimorbidity and mental health cohort study in frailty and aging (MiMiCS-FRAIL): unraveling the clinical and molecular associations between frailty, somatic disease burden and late life depression

**DOI:** 10.1186/s12888-020-02963-9

**Published:** 2020-12-01

**Authors:** Ivan Aprahamian, Ronei Luciano Mamoni, Nilva Karla Cervigne, Taize Machado Augusto, Carla Vasconcelos Romanini, Marina Petrella, Daniele Lima da Costa, Natalia Almeida Lima, Marcus K. Borges, Richard C. Oude Voshaar

**Affiliations:** 1 Geriatrics Division, Department of Internal Medicine, Faculty of Medicine of Jundiaí, Jundiaí, Brazil; 2 Institute and Department of Psychiatry, University of São Paulo, São Paulo, Brazil; 3 University Medical Center Groningen, University Center for Psychiatry and Interdisciplinary Center for Psychopathology of Emotion Regulation, Groningen, Netherlands

**Keywords:** Frailty, Depression, Multimorbidity, Inflammation, Cohort study, Elderly

## Abstract

**Background:**

To explore the mutual relationship between multimorbidity, mental illness and frailty, we have set-up the Multimorbidity and Mental health Cohort Study in FRAILty and Aging (MiMiCS-FRAIL) cohort. At the population level, multimorbidity, frailty and late-life depression are associated with similar adverse outcomes (i.e. falls, disability, hospitalization, death), share the same risk factors, and partly overlap in their clinical presentation. Moreover, these three variables may share a common underlying pathophysiological mechanism like immune-metabolic dysregulation. The overall objectives of MiMiCS-FRAIL are 1) to explore (determinants of) the cross-sectional and longitudinal relationship between multimorbidity, depression, and frailty among non-demented geriatric outpatients; 2) to evaluate molecular levels of senoinflammation as a broad pathophysiological process underlying these conditions; and 3) to examine adverse outcomes of multimorbidity, frailty and depression and their interconnectedness.

**Methods:**

MiMiCS-FRAIL is an ongoing observational cohort study of geriatric outpatients in Brazil, with an extensive baseline assessment and yearly follow-up assessments. Each assessment includes a comprehensive geriatric assessment to identify multimorbidity and geriatric syndromes, a structured psychiatric diagnostic interview and administration of the PHQ-9 to measure depression, and several frailty measures (FRAIL, Physical Phenotype criteria, 36-item Frailty Index). Fasten blood samples are collected at baseline to assess circulating inflammatory and anti-inflammatory cytokines, leukocytes' subpopulations, and to perform immune-metabolic-paired miRome analyses. The primary outcome is death and secondary outcomes are the number of falls, hospital admissions, functional ability, well-being, and dementia. Assuming a 5-year mortality rate between 25 and 40% and a hazard rate varying between 1.6 and 2.3 for the primary determinants require a sample size between 136 and 711 patients to detect a statistically significant effect with a power of 80% (beta = 0.2), an alpha of 5% (0.05), and an R^2^ between the predictor (death) and all covariates of 0.20. Local ethical board approved this study.

**Discussion:**

Frailty might be hypothesized as a final common pathway by which many clinical conditions like depression and chronic diseases (multimorbidity) culminate in many adverse effects. The MiMiCS-FRAIL cohort will help us to understand the interrelationship between these variables, from a clinical perspective as well as their underlying molecular signature.

## Background

In its essence, geriatric medicine is based on comprehensive care, which transfers the idea of integrating social, psychological, biological, and personal/individual factors [1]. This concept is based on the close relationship between these factors and has additive value above traditional disease-based medicine in the health care for older persons [1]. Better understanding of the relationship between these components, as well as their mediating and moderating variables will facilitate person-centered care and shared decision making. In geriatric medicine, multimorbidity, mental illness and frailty are highly prevalent, often interrelated, and all affected by social, psychological and biological factors. To explore their mutual relationship, we have set-up the Multimorbidity and Mental health Cohort Study in FRAILty and Aging (MiMiCS-FRAIL) cohort.

Multimorbidity is defined as the co-occurrence of two or more incurable chronic diseases and affects almost two thirds of older adults [2]. This concept can be viewed as an evolution of comorbidity, in which a determined disease is ranked as being most important or receives more attention. Comorbidity is less suitable to geriatric outpatients who are generally affected by multiple conditions and ranking of these entities remain arbitrary. Multimorbidity is associated with functional disability, lower quality of life and mortality [3–5]. Due to its variable evolution, individualized management is of utmost importance to improve its course, management and prognosis [6, 7]. Moreover, geriatric syndromes (i.e. non-morbid heterogeneous clinical conditions with underlying multifactorial origins, e.g. dizziness, urine incontinence or fatigue) are hardly taken into account in multimorbidity indices, while these syndromes also negatively influence the disease-disease and drug-disease interactions seen in multimorbid states and are by itself associated with a high ‘disease’ burden [8]. Treatment trials involving older adults generally do not stratify participants for relevant geriatric syndromes, adjust data analyses for the presence of geriatric syndromes or specify geriatric syndrome as outcome characteristics. Such knowledge, however, is crucial to select the most appropriate intervention for older adults in routine clinical care [9]. While frailty and mental illness, especially late-life depression, are both considered as geriatric syndromes, both can be hypothesized to mediate and/or moderate the onset and consequences of multimorbidity [8].

Frailty is an overarching concept of biological ageing. Frailty is defined as a biological condition of low homeostatic reserve due to multiple physiologic impairments, which leads to a higher risk of adverse outcomes when exposed to minimal stress [10]. In contrast to other geriatric syndromes, frailty can be subclinical and frail elderly not necessarily have complaints and/or experience disturbing symptoms. Frailty is prevalent around 10% among community-dwelling older persons [11] and an important prognostic marker for adverse health outcomes such as falls, disability, hospitalization and death in all areas of medicine [7, 12]. Two worldwide dominant operationalizations of frailty are 1) the deficit accumulation model (Frailty Index), stating that the proportion of ageing-related deficits reflects biological age on top of chronological age [13, 14] and 2) the Fried Frailty Phenotype [10], which mark an underlying physiologic state of multisystem and energy dysregulation [15]. Several other frailty definitions can be considered as a derivation of one of these models [15].

Mental illness is traditionally treated in mental health care facilities in contrast to somatically oriented hospitals or health care systems. In terms of geriatric psychiatry, depression is one of the most common mental disorders [16]. The prevalence of major depression disorder ranges from 1 to 5% in community-based studies, showing a 2- to 5-fold increase among older adults with multimorbidity [17]. Clinically significant depressive symptoms (aka, minor, subthreshold or subsyndromal depression) is much more frequent among older adults, with a mean prevalence of 15% [18]. Late-life depression has a multifactorial origin in line with the definition of a geriatric syndrome, and is associated with a higher level of multimorbidity, worse prognosis of comorbid chronic somatic disorders, poor cognitive and social functioning, and higher level of self-neglect [16]. Like frailty, late-life depression is an independent predictor of geriatric syndromes (e.g. falls, frailty) as well as disability, hospitalization, institutionalization, and death [19–21]. Several meta-analyses have shown that depression is an independent risk factor for the onset of hypertension, diabetes, stroke, heart disease, Alzheimer’s disease, and cancer [19]. These adverse health outcomes and the association between depression and somatic disorders can only partly be explained by poor healthy behaviors [19, 20, 22]. It is believed that depression shares common pathophysiological mechanisms with physical diseases and geriatrics syndromes such as frailty, including immune-inflammatory-, metabolic-, autonomic nervous system, and hypothalamic-pituitary-adrenal axis dysregulation [19]. Nonetheless, when treatment in primary care stagnates, depressed older persons are generally referred to mental health care facilities outside the setting of general hospital or reach of geriatric medicine. Despite the high disease burden of depressive disorder, depression remains often undiagnosed and untreated in later life [23]. Furthermore, the pathophysiology of late-life depression is less well understood compared to other common chronic somatic diseases [24].

At the population level, multimorbidity, frailty and depression represent a complex clinical combination. All three conditions are associated with similar adverse outcomes (i.e. falls, disability, hospitalization, death), share the same risk factors (e.g., older age, female sex, low income, ethnical background, undernutrition, obesity, functional disability), and partly overlap in their clinical presentation and/or diagnostic criteria [9, 12, 17, 18]. Available prospective studies show bidirectional relations between each other. Meta-analyses show that approximately 40% of depressed older adults can be classified as frail, whereas the other way around, 40% of frail persons can be classified as depressed, whereas the sparse longitudinal studies point to a reciprocal association [25] Longitudinal studies also suggest a bidirectional relation between frailty and multimorbidity. Cross-sectionally, however, frail seniors generally present with multimorbidity (72%), while the inverse relation is not the same (16%) [26]. Collectively, these findings argue for studying multimorbidity, frailty and late-life depression jointly within a longitudinal design. In addition to replicating the assumed bidirectional associations between these conditions, this would also enable to study mediating and moderating effects of multimorbidity, frailty and late-life depression with respect to their mutual adverse health outcomes as well as to study shared underlying pathophysiological mechanisms like immune-metabolic dysregulation.

Immuno-metabolic dysregulation is considered one of the most important changes that occur during ageing [27, 28] and is often associated with multimorbidity of several geriatric syndromes, including frailty and late-life depression [29–31]. Age-related inflammatory processes can be examined at the molecular, cellular as well as systemic level. As these levels are highly interwoven, age-related chronic inflammation should be comprehensively examined. The concept of ‘senoinflammation’ has been recently proposed by Chung and colleagues [32] to provide a comprehensive framework of age-related inflammation integrating these three levels. Senoinflammation ranges from the redox-sensitive core transcription factor NF-κB and polarized macrophages, to non-coding microRNAs (epitranscriptomes) and metabolically linked proinflammatory processes which are not conventionally considered in age-related chronic inflammation [32]. Epigenetics and epitranscriptomes are of particular interest to explain the biological blueprint of social determinants of ageing [33, 34]. Epigenetics refer functionally relevant changes to either the genome (DNA) that do not involve a change in the nucleotide sequence, whereas epitranscriptomes to changes to the transcriptome (RNA) that do not involve a change in the ribonucleotide sequence. Environmental circumstances and social experiences during senescence may result in epigenetic changes with long-lasting biological effects on physiological systems, e.g. the immune system [35], as well as clinical phenotypes of ageing, e.g. frailty [36]. However, data to determine the mechanisms of the consequences of environmental deprivation and social determinants on brain plasticity and mental health in late-life are lacking [35]. In the present study, immune-metabolic dysregulation will be studied according to the concept of ‘senoinflammation’ including epigenetics and epitranscriptomes.

The overall objectives of MiMiCS FRAIL are 1) to explore (determinants of) the cross-sectional and longitudinal relationship between multimorbidity, depression, and frailty among non-demented geriatric outpatients; 2) to evaluate molecular levels of senoinflammation as a broad pathophysiological process underlying these conditions; 3) to examine the long-term consequences of multimorbidity, frailty and depression and their interconnectedness.

## Methods/design

### Study design and participants

The Multimorbidity and Mental health Cohort Study in Frailty and Aging (MiMiCS-FRAIL) is an ongoing prospective observational cohort study of geriatric outpatients in the city of Jundiaí (State of São Paulo, southwestern of Brazil). Eligible persons are all referrals (from primary care facilities or direct access from the community) to an academic-based geriatric outpatient facility that fulfill the inclusion criteria of (a) being 60 years or older, (b) having adherence to regular clinical follow-up (including at least one research visits every 12 months), (c) signing an informed consent for the research. We excluded individuals with conditions that may interfere with reliable data collection, i.e. (1) a diagnosis of dementia; (2) severe mental illness, e.g. bipolar disorder, primary psychotic disorder, or severe substance use disorder in need of detoxification; (3) *delirium* or hospitalization in the last month; (4) electroconvulsive therapy (ECT) in the last 12 months; (5) sensory impairment (vision and hearing); as well as (6) wheelchair users; (7) severe limb paresis or paralysis due to stroke; and (8) unstable clinical conditions, including oncologic patients and terminal illnesses.

The recruitment for this study has started in July 2019. All participants receive an extensive assessment at baseline by a trained staff composed of geriatricians, psychiatrists, physical therapists, nutritionists, and where possible supervised medical residents and graduates. The baseline assessment includes a comprehensive geriatric assessment, a structured psychiatric diagnostic interview, and the use of validated observer-based and self-report questionnaires (see measurements). All participants will be followed-up annually for at least 5 years. These follow-up assessments include all variables amenable to change (see measurements).

Participants can withdraw their consent at any time without giving any reason and without any impact on the clinical care they receive.

### Ethics

The study follows the standards established by the Brazilian National Council of Health. All procedures are conducted in accordance with the ethical standards for research with humans stipulated by the Helsinki Convention. The ethical review boards and local committee (Jundiaí Medical School) approved this study (CAAE numbers 12,535,218.5.3001.5412). MiMiCS-FRAIL is designed as an ongoing project, allowing to replace or add specific measures periodically, provided that these amendments will first be approved by the medical ethical committee. Written informed consent is obtained from all participants and/or their legal guardians.

### Measurements

The main measurements in this cohort study focus on the assessment of somatic disease burden (multimorbidity and geriatric syndromes), frailty and depression, as well as evaluation of senoinflammation as an overall underlying pathophysiological mechanism (Table 1).

**Table 1:** Main measurement of the MiMiCS-FRAIL cohort

Measurement	Baseline	Annually
- Number of chronic diseases (multimorbidity)	x	x
- Number of geriatric syndromes	x	x
- Charlson Comorbidity Index	x	x
- FRAIL questionnaire (self-report Brazilian version)	x	x
- Physical frailty phenotype	x	x
- 36-item Frailty Index	x	x
- Structured Clinical Interview for DSM-5 (section on mood disorders)	x	x
- Patient Health Questionnaire 9-item version	x	x
- Circulating inflammatory and anti-inflammatory cytokines	x	
- Phenotype of major circulating leukocytes subpopulations	x	
- Immuno-metabolic-paired miRome analyses	x	
- Covariates	x	x

#### Somatic disease burden (multimorbidity)

The somatic disease burden is operationalized as two independent variables, i.e. the number of chronic somatic diseases (multimorbidity) as well as the number of geriatric syndromes. Multimorbidity is often defined as the simultaneous presence of two or more chronic diseases. To avoid dichotomization, our primary variable is the number of chronic somatic diseases. This variable is composed by the sum of 10 identified diseases based on history taking, physical examination and complimentary exams. We assess cardiovascular disease (myocardial infarction, atrial fibrillation, heart failure, valvulopathy, coronary artery disease), stroke, diabetes, peripheral vascular disease, lung diseases (asthma & COPD), cancer, chronic renal failure, hepatic disease, osteoarthrosis, thyroid diseases. In addition, we also apply the Charlson Comorbidity Index, a cumulative index combining the number and severity of concurrent diseases [37].

The sum of the number of geriatric syndromes is composed by the presence of the following 12 (“geriatric giants”) as assessed by the comprehensive geriatric assessment: anaemia, cognitive impairment, hearing impairment, visual impairment, sleep disorders, syncope, urine incontinence, osteoporosis, falls, weight loss/anorexia, mobility problems, vertigo.

#### Frailty

Frailty is assessed according to the most commonly used models, i.e. the Frailty Phenotype according to five criteria of Fried and colleagues [10] as well as the short-report FRAIL-BR questionnaire [38] and according to the deficit accumulation model of Rockwood and colleagues [14]. The Fried criteria include weight loss, gait speed, strength, presence of fatigue and physical activity [10]. Weight loss is identified with a positive answer to the question “In the last year, have you lost more than 4.5 kilograms (or > 5%), that is, not due to dieting or exercise?”. Slow gait is defined by an average walking speed ≥7 s for 4.5 m (≥6 s for men > 173 cm and for women > 159 cm). Low muscle strength is evaluated by the average of three handgrip strength measures (in kilograms) in the dominant hand using the Saehan manual dynamometer. Handgrip strength is classified according to cut-offs validated to the Brazilian population according to Table 2. The presence of fatigue is defined by answers (a) “I felt that everything I did was an effort” or (b) “I could not get going” in “a moderate amount of the time” or “most of the time” in the last week. Finally, low physical activity is based on kilocalories expended per week, calculated using the short form of the International Physical Activity Questionnaire [39]. The cutoff values were stratified by sex (men ≤344 kcals and women ≤328 kcals). Older adults meeting none of the five criteria are considered robust, those meeting one or two as prefrail, and finally those meeting three or more criteria are considered frail.

**Table 2:** Cutoff for handgrip strength according to sex and body mass index^a^

Men		Women	
BMI (Kg/m^2^)	Handgrip strength (Kg)	BMI (Kg/m^2^)	Handgrip strength (Kg)
≤ 22,4	≤ 20,0	≤ 23,0	≤ 13,0
22,5 – 24,6	≤ 21,4	23,1 – 26,4	≤ 14,0
24,7 – 27,1	≤ 24,0	26,5 – 29,7	≤ 14,0
> 27,1	≤ 24,0	> 29,7	≤ 14,0

Abbreviations: *BMI* body mass index, *Kg* kilograms; *m*^*2*^ square meters

^a^ According to Nunes DP, Duarte YA de O, Lebrão ML, et al. Screening for frailty in older adults using a self-reported instrument. Rev. Saude Publica. 2015;49:2. 10.1590/S0034-8910.2015049005516

The FRAIL-BR questionnaire is the Brazilian version of the FRAIL [40], a self-report questionnaire mainly based on the Fried Frailty Phenotype. The FRAIL-BR includes the components fatigue, muscle resistance, ambulation, disease burden, and weight loss with the following criteria: (1) Fatigue: the answers “all the time” or “most of the time” to the question “How much of the time during the past 4 weeks did you feel tired?”; (2) Resistance: “yes” to the question “By yourself and not using aids, do you have any difficulty walking up 10 steps without resting?”; (3) Ambulation: “yes” to the question “By yourself and not using aids, do you have any difficulty walking several hundred yards?”; (4) Illness: presence of five or more illnesses out of 11; and (5) Loss of weight: respondents with a weight loss ≥5% of their total weight within 1 year. Each affirmative answer yields 1 point, classifying the person as robust (0 points), prefrail (1–2 points) and frail (3–5 points) [9]. The FRAIL-BR is fast, simples and lack of any specialized training or equipment requirements. The FRAIL-BR is currently recommended for application in clinical settings by international consensus [41] as it facilitates screening and assessment with similar prognostic accuracy compared to more complex instruments [42].

Finally, a well-validated 36-item Frailty Index (FI) is used in this study [43]. The FI is the proportion of health deficits out a count of at least 30 health deficits (symptoms, signs, syndromes, conditions, etc) across different health domains. The FI is a stronger predictor for adverse health outcome than its variables alone [14]. We evaluated 36 health deficits, i.e. anemia, arthritis, cognitive impairment, visual impairment, diabetes, dyspnea, chronic renal disease, sleep disorder, peripheral vascular diseases, urinary tract disorders, thyroid disease, respiratory disease, cerebrovascular disease, ischemic heart disease, atrial fibrillation, fracture, hypertension, syncope, heart failure, urinary incontinence, disability, care dependency, osteoporosis, falls, parkinsonism and related disorders, loss of appetite or anorexia, polypharmacy, foot disorders, mobility problems, obesity, hearing loss, valvopathy, dizziness, social vulnerability, pressure ulcers, peptic ulcers. The FI score can be interpreted as < 0.15 indicative of robustness, 0.15–0.25 as prefrail condition, and a score > 0.25 as frail [43].

#### Depression

Major depressive disorder (MDD) as well as subthreshold depression defined as “another specified depressive disorder” will be assessed according to DSM-5 criteria using the mood section of the Structured Clinical Interview for DSM-5 disorders (SCID-5) [44]. Based on the age of onset of the first major depressive episode, MDD will be classified as early-onset or late-onset using a threshold of 60 years. We also evaluate symptom’s severity with the self-report Patient Health Questionnaire (PHQ) 9-item version [45]. The 9 items of PHQ-9 correspond to the DSM-5 criteria for a depressive episode and have to be rated on a Likert scale ranging from 0 to 3 corresponding to “not at all”, “several days”, “more than half the days” and “nearly every day”. Whether symptoms interfere with daily activities is evaluated in a tenth question. A score between 1 and 4 points is considered normal; 5–9 as mild depression, 10–14 as moderate depression, 15–19 as moderately severe, and 20–27 as severe depression. In 2013, PHQ-9 was validated for the older people in Brazil [46].

#### Senoinflammation

Senoinflammation refers to the comprehensive framework of age-related senescent inflammation at the molecular, cellular as well as systemic level.

##### Low-graded inflammation (inflammaging)

Since the imbalance between pro- and anti-inflammatory responses is thought to be responsible for the development of age-related morbidities [47, 48], an extensive panel of circulating inflammatory and anti-inflammatory cytokines/chemokines will be evaluated in this cohort: IL-6, TNF-alpha, IL-1beta, IL-17A, IL-22, CCL2 (MCP-1), CCL5, CXCL8 (IL-8), CXCL10 (IL-10), IL-27 and IL-37.

##### Immunosenescence

Immunosenescence will be evaluated in a subcohort of 120 individuals as a proof of concept. The phenotype of major circulating leukocytes subpopulations (neutrophils, eosinophils, monocytes, lymphocytes, and NK cells) will be characterized ex vivo by flow cytometry. Additionally, functional evaluation of neutrophils (cytokines - TNF-alpha, IL-6, IL-1beta, IL-10 and Reactive Oxigen Species production and microbicidal activity) and Peripheral Blood Mononuclear Cells (proliferation capability and cytokine production; lymphocytes = IFN-gamma, IL-4, IL-17A, IL-22, IL-9, IL-10; monocytes = TNF-alpha, IL-6, IL-1beta, IL-10, IL-23, IL-12p70) will be analyzed after in vitro stimulation (by ELISA and/or flow cytometry). Inclusion of state-of-the-art parameters for immuno-metabolic dysregulation in line with the concept of ‘senoinflammation’, enables to examine immune-metabolic dysregulation in a breakthrough model accessing multiple pro/antiinflammatory pathways in aging.

##### (Epi) Transcriptomics analyzes

Advances in transcriptomic high-throughput approaches have highlighted significant deregulation of inflammatory genes and proteins during ageing and diseases [49, 50]. Particularly, microRNA expression alterations are the one of multiple epigenetic biomarkers that reflects functional changes in aged subject [51]. Within a subcohort of 90 participants, immuno-metabolic-paired miRome analyses will be performed in plasma and peripheral blood leukocytes to identify a microRNA-based molecular signature of “senoinflammation” specifically associated with frailty, somatic disease burden and late life depression phenotypes. MicroRNAs are highly stable, have high serum concentrations and reflect functional changes in many cellular processes during age [51]. With such relevant regulatory functional role, microRNAs are very promising for future clinical application as robust biomarkers and potential therapeutic targets. This proof of concept transcriptomic investigation will be carried out using array-based systems, and the selected microRNAs-biomarkers will be validated in an independent patient cohort using qRT-PCR technology. All adverse clinical outcomes of interest will be associated with differentially expressed microRNA-biomarkers panel. The data resulting of this study has the ability to link a long-known gap between genes and phenotypes of multimorbidity and mental health in Frailty.

### Covariates

Demographic as well as lifestyle characteristics that could be associated with the presence of chronic somatic diseases, geriatric syndromes, frailty or depression are considered as potential confounders. Therefore, age, sex, years of education, living alone (yes/no), income (estimated wages), social support, body mass index (as indicated by the weight (in kg) of the patient divided by its squared length in meter), polypharmacy, cognitive performance based on the 10-point Cognitive Screening (10-CS) [52], involvement in vigorous physical activity (based on estimated time dedicated to physical activity during the week), self-report regular use of alcohol (how many drinks and type of beverage consumed per week), and finally currently smoking (yes/no) will be measured.

### Outcome variables

The primary outcome variable of our cohort will be death. Date and cause of death will be collected during follow-up (by relatives and/or general practitioner’s information) and checked by the death certificate.

Secondary outcome parameters include:

- number of falls will be assessed over the past 12 months and classified as 0, 1, 2 and 3 or more. The presence of fracture and the description of the event are collected.
- number of hospital admissions will be registered and classified as 0, 1, 2–3, 4 or more. Causes for hospital admission are inquired.
- Functional ability – Functional limitations will be measured with the WHO Disability Assessment Schedule (WHODAS 2.0), a 36-item, self-report scale covering six domains of functioning over the past 30 days. The six domains correspond to the ICF dimensions of activity and of participation and include 1) cognition, 2) mobility, 3) self-care, 4) interpersonal functioning, 5) work-, leisure time-, household-related activities), and 6) participation in society [53].
- Well-being – Well-being will be measured by a single item (“Overall, how satisfied are you with life as a whole these days”) according to OECD Guidelines on measuring subjective well-being [https://www.oecd.org/statistics/oecd-guidelines-on-measuring-subjective-well-being-9789264191655-en.htm] and used in the World Values Surveys. This item can be scored on an 11-point interval scale ranging from 0 “not at all satisfied” through 10 “completely satisfied”.
- Onset of dementia. Since participants will be clinically monitored by a geriatrician on a yearly basis, any sign of cognitive decline, either identified by themselves, their family or the geriatrician, will result in formal diagnostic procedures according to current interdisciplinary guidelines.
- Transition in frailty status and/or severity over time.
- Transition in depression status and/or severity over time.

### Power calculation

**Fig. 1:**
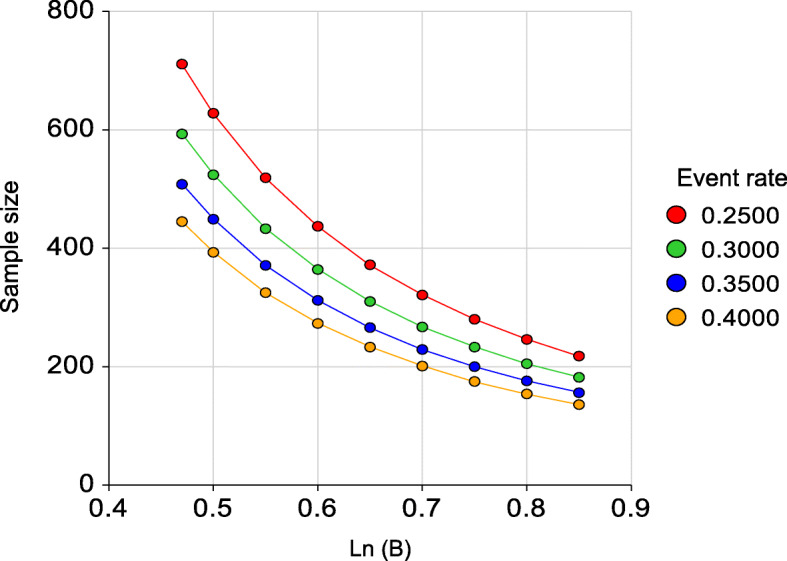
Power calculation of the sample (alpha = 0.05, power = 0.80, R^2^ = 0.20)

The primary outcome parameter (mortality) is estimated between 25 and 40% during a five-year follow-up [12, 54–56]. Based on meta-analyses, the HR (95% CI) of dichotomous measures of either frailty, multimorbidity (2 or more diseases versus no morbidity) and depressive disorder is estimated at 2.34 (1.77–3.09), 1.73 (1.41–2.13), and 1.60 (1.37–1.86), respectively [12, 54–56], corresponding to a regression coefficient of B ranging from Ln (1.60) = 0.47 through Ln (2.34) = 0.85. Assuming a power of 80% (beta = 0.2), an alpha of 5% (0.05), and an R^2^ between the predictor and all covariates of 0.20 (to adjust for covariates as well as examining mediating effects), requires a sample size between 136 and 711 participants to detect a statistically significant effect (see Fig. 1). The use continuous measures of frailty, multimorbidity and depression as predictor variables in Cox-regression analyses and the use of Generalized estimating equation (GEE) or linear mixed models (LMM) analyses for repeated (outcome) measures, will further increase the statistical power and allow for testing mediating effects of senoinflammation and exploration of moderating effects between the three predictor variables (frailty, multimorbidity and depression).

### Statistical analysis

All collected data are stored physically and computerized using both hardware and cloud storage, both encrypted, at the Faculty of Medicine of Jundiaí (Geriatrics Division, Department of Internal Medicine), and can be accessed only by two researchers (IA, CVR). All data will be verified for typos or mismatches every month by a third researcher.

Participants will be classified according to target variables as depressed or not, presenting frailty or not (FI), being robust, pre-frail or frail (FRAIL and frailty phenotype, separately), by the number of diseases or presence of multimorbidity, and by the number of geriatrics syndromes. The characterization of the sample will proceed through descriptive statistics of categorical (chi-square test) and continuous (t test) variables described before. Non-normally distributed continuous variables will be transformed if needed for parametric testing.

The primary outcome parameters (death) will be examined by Cox-regression analyses and adjusted for covariates with time to death as the dependent variable and with multimorbidity, frailty and/or depression (and if relevant their interaction) as independent variables. Indices of seno-inflammation will be explored as mediating variables.

Generalized estimating equation or linear mixed models will be used to evaluate associations depression, somatic disease burden, frailty over time and the mediating role of senoinflammation, adjusted for potential confounders (covariates). Separate models will be built for the different definitions of depression (according to DSM-5 criteria or depressive symptom severity by PHQ-9) and frailty definitions (FRAIL-BR, frailty phenotype, FI).

The software SPSS version 25 or later and R version 3.6.2 or later will be used to statistical analyses. Statistically significant findings are set as type I error of less than 5%.

## Discussion

This cohort study is unique in its design by measuring three important variables in geriatric care, namely multimorbidity, frailty and depression jointly over time. Better knowledge of the mutual relationships between multimorbidity, frailty and depression and shared pathophysiological mechanisms may improve clinical diagnosis and facilitate personalized treatment. Moreover, these data enable us to examine senoinflammation as a shared underlying pathophysiological mechanism as discussed below.

### Senoinflammation

Many systemic, cellular and molecular mechanisms of senescence have been identified [57, 58]. During self-limiting processes, senescent cells are usually removed from tissues by immune-mediated clearance, which restores the physiologic capacity of different tissues and resumes its homeostatic capacity. The enduring of unresolved recurrent senescence pushing over time can lead to the accumulation of senescent cells in different tissues, generating important harmful effects. At the biological level, this scenario can manifest as a reduced capacity to recover from stressors, triggering systemic changes such as lipodystrophy, metabolic dysfunction, vascular hyporeactivity [59]. At the clinical level, senescence can manifest as frailty, geriatric syndromes like cognitive decline, sarcopenia, reduced mobility [60] as well as late-life depression and the onset of many chronic somatic diseases [28].

Immunosenescence affects the quality of the immune response which may result in a chronic systematic inflammatory state also called inflammaging [27, 28]. Inflammaging is considered an adaptation of the immune system to the physiological changes common to the aging process [28]. The imbalance between pro- and anti-inflammatory responses, rather than the inflammatory process itself, has been hypothesized to be responsible for the development of age-related morbidity [48, 55]. Inflammatory dysfunction has indeed been consistently associated with geriatric depression, frailty and multimorbidity [30, 31].

Age-related inflammatory processes can be examined at molecular, cellular as well as systemic level [51]. As these levels are highly interwoven, we will examine age-related chronic inflammation comprehensively as proposed by the concept of ‘senoinflammation’. This concept has been recently proposed by Chung and colleagues to provide a comprehensive framework of age-related inflammation integrating the three levels, which ranges from the redox-sensitive core transcription factor NF-κB and polarized macrophages, to non-coding microRNAs (epitranscriptomes) and metabolically linked proinflammatory processes, and are not conventionally considered in age-related chronic inflammation [51].

Lately, the phenomenon of developmental plasticity, influenced by the environment, has been explored in the science of epigenetics, which has presented novel molecular mechanisms to underlie aging-related previous observations [32]. MicroRNAs are the one of multiple epigenetic biomarkers that reflects functional changes in aged subject [51]. Advances in transcriptomics have highlighted significant deregulation of inflammatory genes and proteins during aging and diseases [50]. These changes are regulated at different levels, including post-transcriptional gene expression regulation by microRNAs. Also, plasma circulating microRNAs have been identified in the context of senescence, ageing and age-associated diseases [51]. Non-coding microRNAs comprise a highly conserved family of small RNAs that generally serve as negative post-transcriptional epigenetic regulators of gene expression. They are predicted to regulate the expression of more than 50% of human protein-coding genes acting through mRNA destabilization and/or translational repression; controlling several cellular biological processes [61]. Plasma circulating microRNAs have been recently identified in the context of senescence, ageing and age-associated diseases [62–67]. Altered expression of the microRNAs may thus contribute to deregulation of the inflammatory/anti-inflammatory balance, promoting aging-associated phenotypes. To what extent these mechanisms explain the interrelationship between multimorbidity, frailty and late-life depression has not been examined yet.

### Methodological considerations

Studies in geriatric medicine are generally prone to attrition bias. Therefore, we have chosen for a clinical, outpatient cohort of older adults receiving long-term, outpatient care at our clinic in the city of Jundiai. This geriatric outpatient clinic serves a relatively well developed class of older individuals. On the one hand, attrition rates will be favorable since participants have a high level of commitment for additional research assessment in conjunction with routine clinical care. On the other hand, generalizability may be compromised by having a lower proportion of less developed persons. Furthermore, embedding the study in an academic environment offers the opportunity for extensive training and supervision of assessors.

Secondly, the study has been set-up as an ongoing cohort since the statistical power for detecting falls, hospitalizations and death is largely dependent on the number of events over time. Although the statistical power is sufficient to detect statistically significant effects in subgroups from 136 older adults onwards, we have the possibility to increase our sample size over time. Being interested in the interaction between multimorbidity, frailty and late-life depression, we aim to include at least 600 persons and a minimum follow-up of 5 years.

Finally, measures of senoinflammation can be affected by caloric restriction and we have not included calorie intake in our protocol.

## Conclusion and clinical implications

It is worth to note that at the population level, multimorbidity, frailty and depression represent a complex clinical combination with bidirectional relations between each other. Findings of the MiMiCS FRAIL cohort study will help to better distinguish between these overlapping syndromes in clinical practice. Despite robust clinical and epidemiological evidences that late-life depression is associated with an enhanced aging phenotype, underlying mechanisms are not well understood [47]. Frailty has a multifactorial origin and probably all ageing-related changes may contribute to the onset of frailty. Frailty might thus be hypothesized as a final common pathway by which many clinical conditions like depression and chronic diseases culminate in many adverse effects. For example, frailty has been identified as an explanatory factor for the association between depression and multimorbidity among depressed outpatients [68]. The MiMiCS-FRAIL cohort will help us to understand the interrelationship between multimorbidity, frailty and late-life depression, from a clinical perspective as well as their underlying molecular signature. Knowledge on the underlying molecular signature of different combinations of multimorbidity, frailty and depression, may guide future treatment trials and better personalized medicine.

## Data Availability

Researchers interested in collaboration and/or using data of MiMiCS-FRAIL to answer a specific research question can directly contact one of the principal investigators (dr. I. Aprahamian or dr. R.C. Oude Voshaar).

## Abbreviations

COPD: Chronic obstructive pulmonary disease
DSM-5: Diagnostic and Statistical Manual of Mental Disorders, Fifth Edition
FI: Frailty Index
FRAIL-BR: FRAIL questionnaire Brazilian version
IFN: Interferon
IL: Interleukin
MDD: Major depressive disorder
mRNA: RNA messenger
NK: Natural killer cells
TNF: Tumor necrosis factor
